# Malignant Hypertension Mimicking Pulmonary-Renal Syndrome

**DOI:** 10.7759/cureus.80654

**Published:** 2025-03-16

**Authors:** Shannon M Lee, Aida F Martinez, Nathan Yee

**Affiliations:** 1 Department of Internal Medicine, Harbor University of California Los Angeles Medical Center, Torrance, USA; 2 Department of Pulmonary and Critical Care, Harbor University of California Los Angeles Medical Center, Torrance, USA

**Keywords:** glomerulonephritis, hypertension-malignant, lung disease, pulmonary-renal syndrome, pulmonology

## Abstract

Pulmonary renal syndrome (PRS) is a rare clinical syndrome characterized by pulmonary hemorrhage and rapidly progressing glomerulonephritis. It is commonly due to a rheumatologic etiology, including antineutrophil cytoplasm antibodies vasculitis or antiglomerular basement membrane disease. Given the rapid progression, patients are often empirically treated when there is high clinical suspicion for PRS. Few case reports have shown malignant hypertension (HTN) as a mimicker of PRS. We present a case of a patient with dyspnea, hemoptysis, and hematuria where there was high suspicion for PRS, and immunosuppression was initiated, but in the end, the etiology was malignant HTN. Malignant HTN can lead to multiple end-organ damage, including the lungs and kidneys; thus, it is important to consider malignant HTN in the differential. Overall, we report a case of malignant HTN mimicking PRS.

## Introduction

Pulmonary renal syndrome (PRS) is characterized by pulmonary hemorrhage and rapidly progressing glomerulonephritis, often presenting with hemoptysis, acute kidney injury (AKI), and hematuria [1,2]. PRS is most often due to autoimmune conditions, including antineutrophil cytoplasm antibody (ANCA) vasculitis, antiglomerular basement membrane (GBM) disease, and systemic lupus erythematosus [1-3]. The main pathology behind PRS is inflammation and necrosis of the capillaries. In the lungs, destruction of the pulmonary capillaries leads to vessel wall breakdown and necrosis, which can present as hemoptysis. In the kidneys, there is often necrotizing glomerulonephritis, which often presents as hematuria [4]. Given that respiratory and renal compromise may occur rapidly, prompt recognition is vital. Definitive diagnosis of PRS consists of lung biopsy with findings of small vessel vasculitis or renal biopsy with the presence of glomerulonephritis. However, this sometimes may not be feasible in the setting of acute illness [5]. While not diagnostic, bronchoalveolar lavage (BAL) is often performed to confirm diffuse alveolar hemorrhage (DAH) [5]. When suspicion is high, immunosuppressive agents and plasma exchange are initially used, even in cases when the diagnosis is not yet confirmed, as there is a risk of rapid progression [1].

This article was previously presented as a rapid-fire case report presentation at the 2024 CHEST Annual Scientific Meeting on October 7, 2024.

## Case presentation

**Figure 1:**
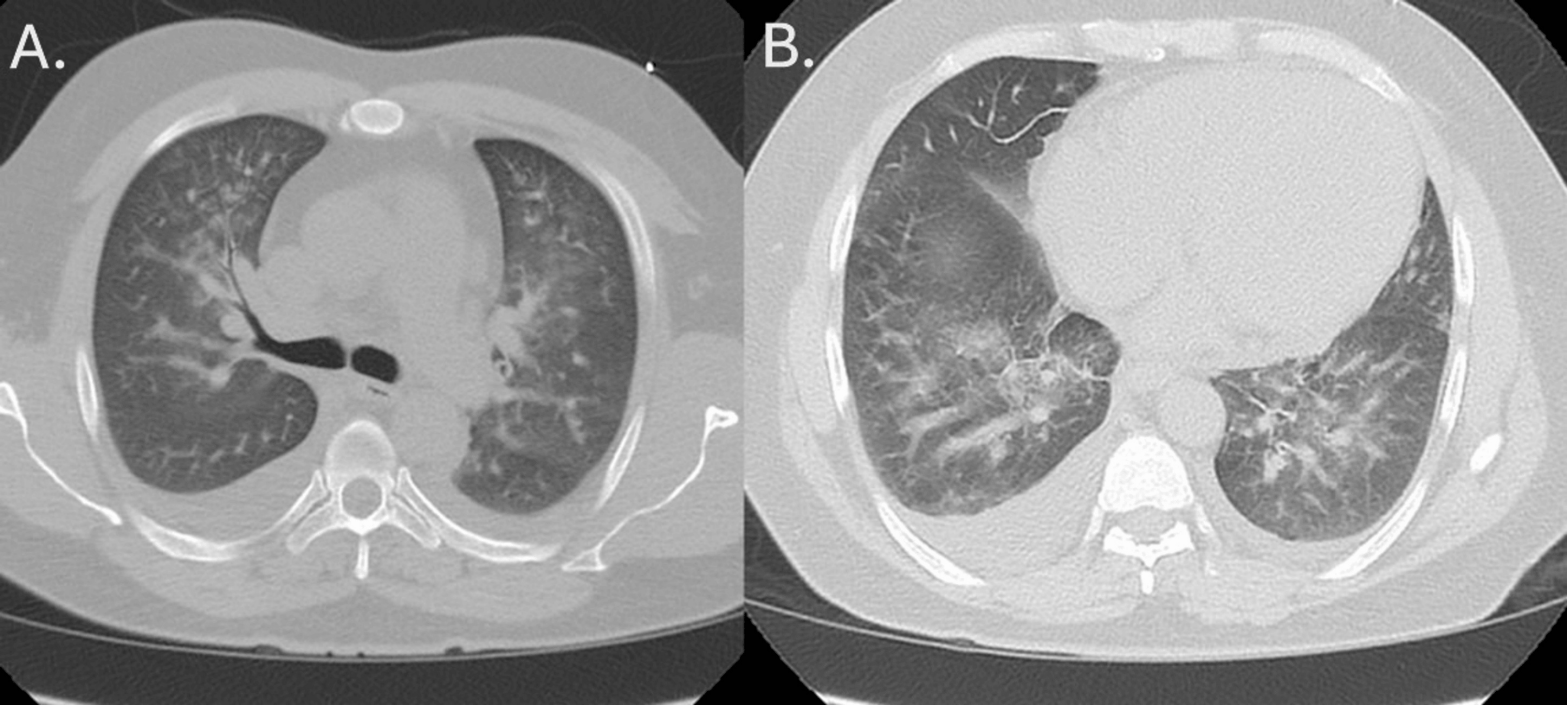
CT of the chest with bilateral ground glass opacities and infiltrates seen at two locations: at the level of the pulmonary arch (A) and below the pulmonary arch (B) CT: computed tomography

A 36-year-old man with a history of hypertension (HTN) presented with two weeks of dyspnea, non-life-threatening intermittent hemoptysis, and hematuria. He had recently stopped his blood pressure medications, which included amlodipine, hydrochlorothiazide, and valsartan, a few months before admission after moving from Central America. He had a temperature of 37.3°C, heart rate of 118 bpm, respiratory rate of 25 breaths/minute, and blood pressure of 235/177 mmHg. Laboratory analysis revealed an elevated creatinine of 3.05 mg/dL and a urinalysis (UA) notable for a large amount of blood with 26-50 red blood cells (RBCs)/high-power fields and >600 mg/dL protein. The urine protein/creatinine ratio was 4.06. His blood pressure quickly improved after receiving IV labetalol in the emergency department. Given high concern for pulmonary-renal syndrome based on presentation, a broad autoimmune workup including ANCA, antimyeloperoxidase (MPO), antiproteinase-3 (PR3), and GBM was sent out. Computerized tomography of the chest demonstrated bilateral ground glass opacities and infiltrates (Figure 1).

**Figure 2:**
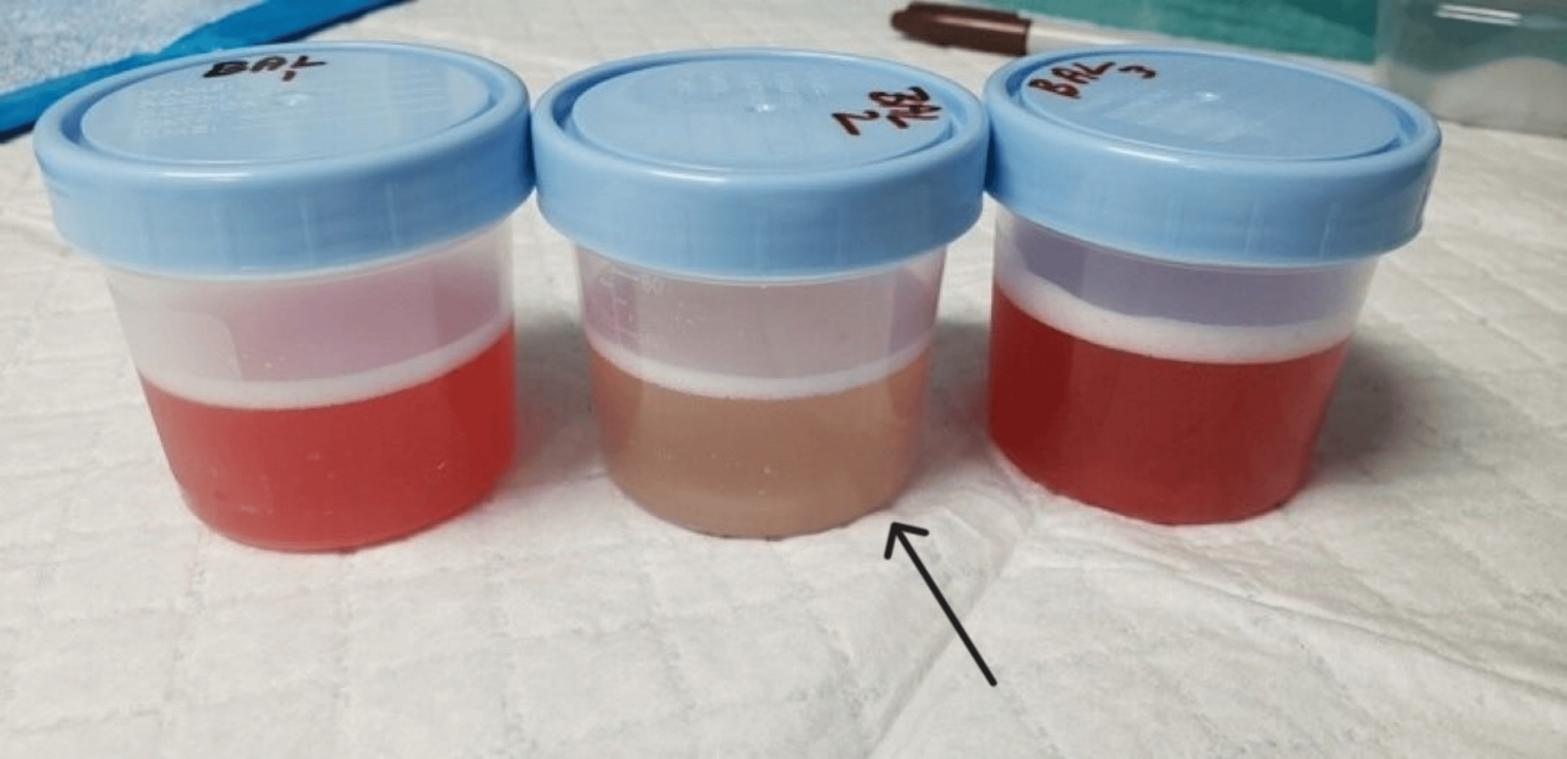
BAL results showing bloody return (right and left). Subsequent aliquot (middle) does not consistently show hemorrhagic return BAL: bronchoalveolar lavage

During his hospitalization, the patient's blood pressure management was escalated to include carvedilol and nifedipine. Rheumatology and nephrology were consulted, and due to high concern for rheumatologic etiology, pulse-dose steroids and immunosuppressive therapy were initiated. In addition, the pulmonology team evaluated the patient, and a bronchoscopy with BAL was performed in the right middle lobe, lingula, and left lower lobe. BAL fluid was bloody, but serial aliquots of BAL fluid did not consistently show an increasingly hemorrhagic return consistent with bland pulmonary hemorrhage rather than DAH (Figure 2). In addition, while hemoptysis was documented before the bronchoscopy with BAL, no hemoptysis was documented after. Infectious workups, including BAL studies, later returned negative results. Before discharge, autoimmune workup, including ANCA, MPO, PR3, and GBM, returned negative, leading to the discontinuation of pulse-dose steroids and immunosuppressive therapy. The patient was discharged with antihypertensive medications and steroid taper, as well as a plan for a renal biopsy.

**Figure 3:**
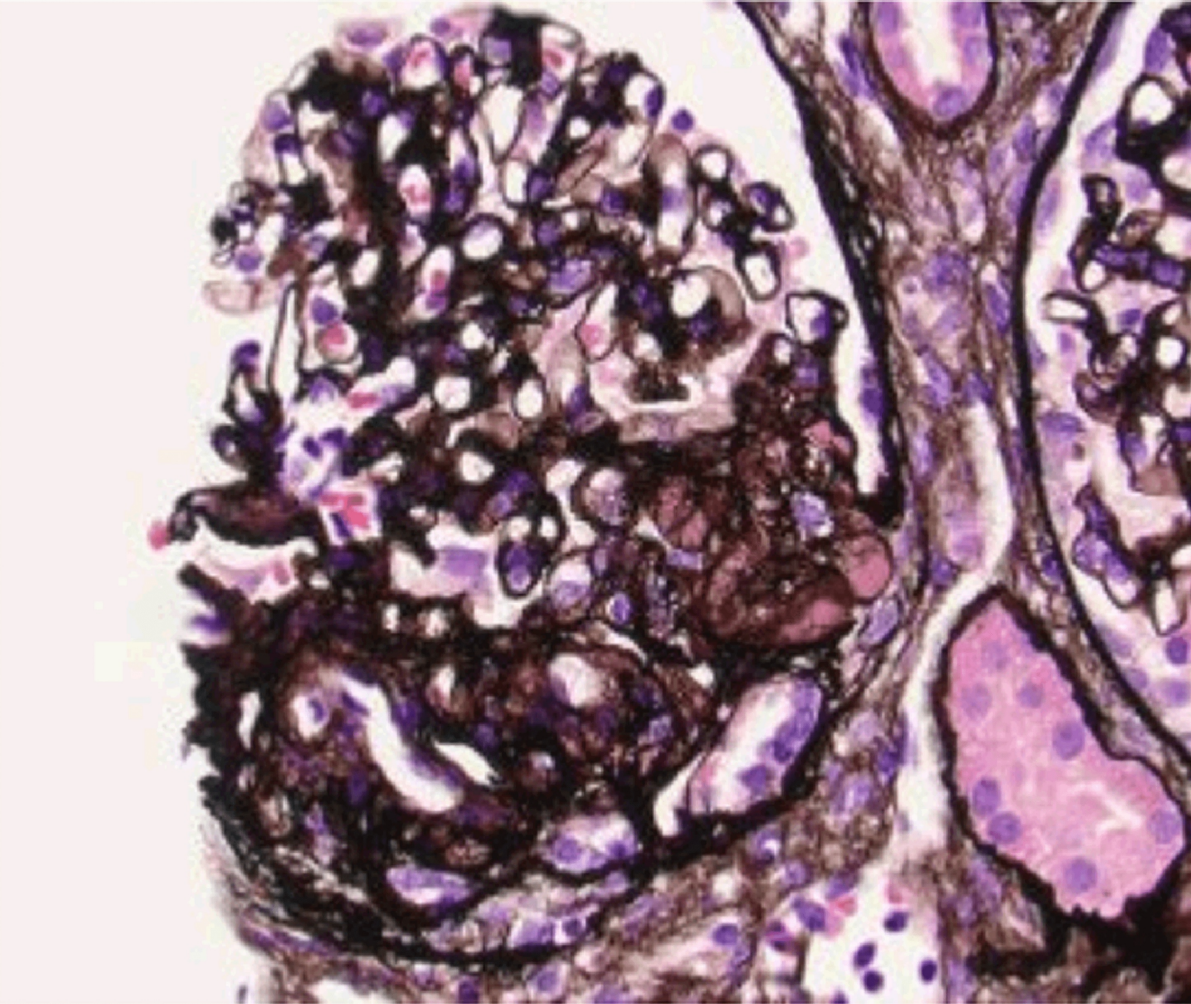
Renal biopsy results with JMS stain to highlight the segmental glomerulosclerosis JMS: Jones methenamine silver

Two weeks after discharge, he underwent a renal biopsy, which showed thrombotic microangiopathy (TMA) with secondary focal and segmental glomerulosclerosis and acute tubular injury (Figure 3). Findings were consistent with acute injury from malignant HTN in the setting of chronic uncontrolled HTN. There was no evidence of glomerulonephritis or vasculitis. Ultimately, malignant HTN was the etiology of his hemoptysis and hematuria with AKI. Four months after discharge, he was adherent to his antihypertensive regimen and normotensive with some improvement in renal function to serum Cr 2.69 mg/dL and resolution of hematuria given trace protein seen on UA.

## Discussion

PRS consists of the combination of DAH and rapidly progressive glomerulonephritis. PRS is most commonly due to autoimmune conditions: 70% of cases are due to ANCA vasculitis, and 20% are due to anti-GBM disease [2]. The remaining 10% of cases are attributed to rheumatoid arthritis, mixed connective tissue disease, and poststreptococcal glomerulonephritis [2]. The pathological process for PRS differs based on the condition, but it is commonly due to small vessel inflammation in the pulmonary capillaries and the glomerulus. In the lungs, the inflammation in the capillaries leads to neutrophil influx, which disrupts the integrity of the capillaries, allowing for RBCs to flood into the alveoli [2]. This presents as DAH. The renal pathology consists of fibrinoid deposition and crescentic inflammation, ultimately causing glomerulonephritis, which often presents as hematuria [6].

Malignant HTN is defined as elevated blood pressure with target end-organ damage, including the brain, eyes, and kidneys. This may present as acute encephalopathy, retinopathy, kidney injury, and/or hematuria [7,8]. Increased blood pressure results in mechanical stress and widespread endothelial dysfunction, leading to capillary leakage and hyperperfusion with disruption of pressure regulation in the arterioles and development of TMAs [8,9]. Unfortunately, the diagnosis of malignant HTN is often missed and only established once there is end-organ damage [10]. The absence of treatment can lead to poor prognosis, so identification and blood pressure management are key. Malignant HTN can have substantial long-standing effects if not treated.

Malignant HTN can affect multiple organ systems, including the pulmonary and renal systems. Malignant HTN causes increased permeability of the endothelium, which can lead to fibrin deposits, smooth muscle necrosis, and vascular injuries at the capillary level [9]. In the pulmonary system, HTN acts through small vessel and capillary inflammation, leading to fibrinoid necrosis. On renal histology, there can be ischemic retraction of the glomeruli as well as small artery lesions like concentric medial smooth muscle hypertrophy, also known as onion skinning [9]. Due to damage of the capillaries, RBCs cross the capillary membrane and enter the alveoli [1,2,11]. On BAL, there is often some blood; however, this contrasts with PRS, where there is DAH, which presents with progressively bloody aspirates from the BAL [2]. In the kidneys, HTN presents as fibrinoid necrosis of the afferent arterioles and proliferative endarteritis of the arteries, narrowing the arterial lumen and causing ischemia [1]. This contrasts with PRS, where kidney biopsy presents as glomerulonephritis. Unfortunately, 63% of patients who present with malignant HTN already have renal impairment [1]. In patients with end-organ damage in the renal system, renal recovery is often poor at five years, with a risk of dialysis dependence despite adequate blood pressure control [6,10]. Overall, early identification of high blood pressure and management with lifestyle changes and oral hypertensives is critical in preventing downstream end-organ effects.

Only a handful of cases of malignant HTN mimicking PRS are documented in the literature. Park et al. [7] reported a patient with no history of HTN presenting with HTN, hemoptysis, and elevated creatinine levels, accompanied by microscopic hematuria and proteinuria. This patient was initially started on steroid therapy before a negative immunological workup. In addition, Yong and Power [1] report a patient with an almost decade-long history of uncontrolled HTN presenting with hemoptysis, headache, and epigastric pain; in this case, he was treated with steroids, cyclophosphamide, and plasma exchange before a negative immunological workup. While this combination of pulmonary and renal damage is often due to immunological conditions, it is important to consider malignant HTN in the diagnosis. This case illustrates how malignant HTN can lead to end-organ damage in the lungs and kidneys, mimicking a rare diagnosis like PRS.

## Conclusions

This case highlights the importance of keeping malignant HTN in the differential diagnosis, especially when the patient presents with uncontrolled HTN. Recognition and management of malignant HTN is crucial in preventing further end-organ damage. Overall, this case illustrates how malignant HTN can lead to multiple end-organ damage to mimic a rare diagnosis like PRS. This is important because there are only a handful of cases of malignant HTN mimicking PRS.
